# A missense variant in *IFT122* associated with a canine model of retinitis pigmentosa

**DOI:** 10.1007/s00439-021-02266-3

**Published:** 2021-02-19

**Authors:** Maria Kaukonen, Inka-Tuulevi Pettinen, Kaisa Wickström, Meharji Arumilli, Jonas Donner, Ida-Julia Juhola, Saila Holopainen, Joni A. Turunen, Masahito Yoshihara, Juha Kere, Hannes Lohi

**Affiliations:** 1 Department of Veterinary Biosciences, University of Helsinki, Helsinki, Finland; 2 Department of Medical and Clinical Genetics, University of Helsinki, Helsinki, Finland; 3 Folkhälsan Research Center, Helsinki, Finland; 4 Veterinary Clinic Kamu, Oulu, Finland; 5 Genoscoper Laboratories Ltd (Wisdom Health), Helsinki, Finland; 6 Department of Equine and Small Animal Medicine, University of Helsinki, Helsinki, Finland; 7 Department of Ophthalmology, University of Helsinki, Helsinki University Hospital, Helsinki, Finland; 8 Department of Biosciences and Nutrition, Karolinska Institutet, Huddinge, Sweden; 9 Stem Cells and Metabolism Research Program STEMM, University of Helsinki, 00014 Helsinki, Finland

## Abstract

Retinitis pigmentosa (RP) is a blinding eye disease affecting nearly two million people worldwide. Dogs are affected with a similar illness termed progressive retinal atrophy (PRA). Lapponian herders (LHs) are affected with several types of inherited retinal dystrophies, and variants in *PRCD* and *BEST1* genes have been associated with generalized PRA and canine multifocal retinopathy 3 (cmr3), respectively. However, all retinal dystrophy cases in LHs are not explained by these variants, indicating additional genetic causes of disease in the breed. We collected DNA samples from 10 PRA affected LHs, with known *PRCD* and *BEST1* variants excluded, and 34 unaffected LHs. A genome-wide association study identified a locus on CFA20 (*p*_raw_ = 2.4 × 10^–7^, *p*_Bonf_ = 0.035), and subsequent whole-genome sequencing of an affected LH revealed a missense variant, c.3176G>A, in the intraflagellar transport 122 (*IFT122*) gene. The variant was also found in Finnish Lapphunds, in which its clinical relevancy needs to be studied further. The variant interrupts a highly conserved residue, p.(R1059H), in IFT122 and likely impairs its function. Variants in *IFT122* have not been associated with retinal degeneration in mammals, but the loss of ift122 in zebrafish larvae impaired opsin transport and resulted in progressive photoreceptor degeneration. Our study establishes a new spontaneous dog model to study the role of IFT122 in RP biology, while the affected breed will benefit from a genetic test for a recessive condition.

**Supplementary Information:**

The online version contains supplementary material available at 10.1007/s00439-021-02266-3.

## Introduction

Retinitis pigmentosa (RP) is the most common group of inherited retinal degenerations affecting 1 in 4000 people worldwide (Verbakel et al. 2018). RP is characterized by progressive loss of vision resulting from abnormalities in rod and cone photoreceptor cells or the retinal pigment epithelium (RPE). Most cases are isolated, but approximately 20–30% of RP patients have accompanying extra-ocular manifestations (Ferrari et al. 2011). According to the Retinal Information Network RetNet (http://sph.uth.edu/retnet/, accessed in November 2020), over 70 genes and loci have been implicated in nonsyndromic RP to date, with the majority of them associated with autosomal recessive inheritance (Daiger et al. 1998). Still, many RP patients have a disease of an unknown genetic cause, highlighting the need for new genetic studies and discoveries (Daiger et al. 2013). As we move to the era of personalized medicine with successful gene therapy and gene editing, the knowledge of the causal variants becomes increasingly important.

The domestic dog (*Canis lupus familiaris*) has become a powerful model to study genetics of human inherited diseases over the past 15 years. Currently, more than 280 likely causal variants have been reported to Mendelian traits or disorders in dogs (Online Mendelian Inheritance in Animals database; www.OMIA.org). The discoveries have been facilitated by the unusual genomic architecture of the dog breeds, the abundance of naturally occurring inherited diseases in different dog breeds, improved genomic tools, and expanding dog DNA banks worldwide (Lindblad-Toh et al. 2005; Lequarré et al. 2011; Groeneveld et al. 2016; Hytonen and Lohi 2016; Bunel et al. 2019).

As an example, over 100 dog breeds are affected with progressive retinal atrophy (PRA), an inherited retinal degeneration similar to RP (Miyadera et al. 2012). Findings in ophthalmoscopic examinations of PRA affected dogs typically include diffuse tapetal hyperreflectivity resulting from retinal thinning, attenuation of the retinal blood vessels, and at later stages, atrophy of the optic nerve head (Parry 1953). Over 20 genes have been implicated to date, with the most recent reported from the Lhasa Apso dogs with a LINE-1 insertion variant in the *IMPG2* gene (Miyadera et al. 2012; Hitti-Malin et al. 2020). Another PRA affected breed is the Lapponian Herder (LH), in which the autosomal recessive *PRCD* variant explains a subset of cases (Zangerl et al. 2006). The LH breed also suffers from another inherited retinal disorder termed canine multifocal retinopathy (cmr3), a canine equivalent of bestrophinopathy that results from *BEST1* variants (Zangerl et al. 2010). Typical clinical findings in cmr3 include multiple elevated subretinal brown-gray lesions with fluid accumulation, but generalized PRA has also been proposed as a rare outcome in some older dogs (Zangerl et al. 2010). A third genetic form of inherited retinopathy is suspected in LH, as not all cases of inherited retinal dystrophies have been explained by the known *PRCD* and *BEST1* variants.

With a series of clinical, genetic, and bioinformatics studies, we describe here a recessive missense variant in *IFT122* as the likely cause of PRA in LH. IFT122 represents a novel candidate gene for mammalian RPs.

## Materials and methods

### Study cohort

Samples from 563 purebred LHs and 577 FLs, donated to our canine biobank at the University of Helsinki, were included in this study. The initial discovery cohort consisted of 44 LHs and the breed screening cohort of 519 LHs, respectively. All dogs in the initial cohort had undergone thorough eye examinations performed by veterinary ophthalmologists board-certified by the European College of Veterinary Ophthalmologists. The examination included basic neuro-ophthalmic assessment followed by slit-lamp biomicroscopy for adnexa and anterior segment evaluation and indirect ophthalmoscopy to examine the fundus. Mydriasis was achieved using topical tropicamide (Oftan Tropicamid 1%, Santen, Tampere, Finland). The inclusion criterion for cases was generalized PRA, while controls were examined healthy at the age of ten years or older. EDTA blood samples were collected from all the dogs and genomic DNA extracted from the white blood cells using the semi-automated Chemagen extraction robot (PerkinElmer Chemagen Technologie GmbH, Baeswieler, Germany) following the manufacturer’s instructions. DNA concentration and purity were assessed by using the Qubit fluorometer (Thermo Fisher Scientific, Waltham, MA, USA) and Nanodrop ND-1000 UV/Vis Spectrophotometer (Nanodrop Technologies, Wilmington, DE, USA) and samples were stored at − 20 °C.

### Pedigree analysis and genome-wide association study

A pedigree was drawn around the affected individuals in the initial cohort with the GenoPro 2.5.4.1 software. To perform GWAS, samples from eight cases and 12 controls were genotyped by using the Illumina’s CanineHD BeadChip array with 220,853 markers (San Diego, CA, USA) at the GeneSeek Laboratory (Neogen Genomics, Lincoln, NE, USA). All genotyped samples had a call rate of > 95%. Only markers with > 95% call rate and minor allele frequency of > 5% were included in the analysis, resulting in the total number of 145,387 markers. No markers were removed due to Hardy–Weinberg exact test (threshold *p* = 0.0001). Allele frequency differences between cases and controls were calculated using the Plink v1.90 software (Chang et al. 2015) and multiple testing correction implemented by using the Bonferroni method, which set the genome-wide significance level to 3.4 × 10^–7^. MDS plot and QQ plot analysis and genomic inflation factor lambda were utilized to assess population stratification. The CanFam 3.1 reference genome assembly was used in all genetic studies.

### Whole-genome sequencing

Sample of an affected LH was whole-genome sequenced using the Illumina HiSeq X ultra-high-throughput sequencing platform with paired-end reads (2 × 150 bp) at the Novogene Bioinformatics Institute (Beijing, China). The reads were aligned to dog reference genome CanFam3.1 using the SpeedSeq open-source software with Burrows-Wheeler Aligner (BWA) (v0.7.17), sorting and BAM compression was done using the Sambamba and duplicate reads were marked using SAMBLASTER (Chiang et al. 2015). Post-alignment processing included local realignment around known INDELs and base quality scores recalibration to reduce erroneous variant calls with Genome Analysis Tool Kit GATK (McKenna et al. 2010). Variant calling was done using the HaplotypeCaller in gVCF mode. CombineGVCs was used to combine gVCFs and joint genotyping was done with GenotpeGVCFs using GATK version 4.1.0. Mobile element insertions were analyzed using the Mobile Element Locator Tool (MELT) (Gardner et al. 2017), with reference sequences of the transposons for MEI discovery retrieved from the Repbase database (Jurka et al. 2005). Structural variants were identified with the DELLY2 software (Rausch et al. 2012). Insertions, deletions, duplications and inversions were called independently in DELLY2. Functional annotation of the variants was done in ANNOVAR using Ensembl release-100 and NCBI *Canis lupus familiaris* Annotation Release 105. Variant filtering was performed using webGQT server deployed on dog variant datasets, assuming recessive mode of inheritance, i.e. the case was set to be homozygous for the alternative allele in SNV filtering and controls wild-type (Arumilli et al. 2020). When filtering the SVs and MEIs, the case was allowed to be either homozygous or heterozygous for the alternate allele to prevent erroneous zygosity calling hampering the results, while controls were wild-type. Variant data from exome (*n* = 100) and whole-genome sequenced (*n* = 227) dogs from our other ongoing projects without the studied phenotype and from various breeds were used in filtering as controls (Online Resource 1). Filtering the SNV variants included all 327 dogs, while only the whole-genome sequenced individuals could be utilized in MEI and SV filterings.

### Variant screening

We analyzed *PRCD*-PRA and cmr3 variants, known to affect LHs, in all cases before their inclusion in any further genetic analysis to exclude known inherited retinal disease variants as a cause of disease (Zangerl et al. 2006, 2010). Primer sequences were obtained from the original study for cmr3 (Zangerl et al. 2010) and designed with Primer3 software for the *PRCD* variant (Koressaar and Remm 2007) and were, in 5′ > 3′ orientation, TCCTAATCCAGTGGCAGCAG (F) and ATCAGCTTCTCACGGTTGGA (R) for the *PRCD* variant and AAGGAGGGAAAAGATAGGGT (F) and AGGTGGAAGGAGGGTAGAAT (R) for the two cmr3 variants. Initial segregation analysis for the *IFT122* variant was made using PCR primers TCACCTTGTCCTATGAGCCC (F) and GCAGAGAAGACGAATGGCTG (R), designed with Primer3 software. All PCR products were amplified using Biotools DNA Polymerase (Biotools B&M Labs, S.A., Valle de Tobalina, Madrid, Spain) and treated with exonuclease I and shrimp alkaline phosphatase before capillary sequencing in the Institute for Molecular Medicine Finland (Helsinki, Finland). The sequence data were analyzed using Unipro UGENE v35.0 (UniPro, Novosibirsk, Russia). Variant screening in the LH breed cohort and additional FLs was performed using Custom TaqMan SNP Genotyping Assay (ThermoFisher Scientific, Waltham, MA, USA) and CFX96 Touch Real-Time PCR Detection System (BioRad, Hercules, CA, USA) with the following primers, in 5′ > 3′ orientation, TGTGGTTCTTGTGCTTTTGTAGGA (F) and TGCCCAGGTTGTTCAGCAA (R) and probes VIC-TGCTACCGCTGCTCC (wild-type) and FAM-TGCTACCACTGCTCC (variant). Finally, large-scale screening of the *IFT122* variant was performed in a sample set of 6023 dogs from 183 breeds and 130 mixed-breed dogs (Online Resource 2) submitted for commercial testing at Genoscoper Laboratories Oy, Helsinki, during 2019–2020. Genotyping in this sample set was carried out according to manufacturer-recommended standard protocols on a custom-designed Illumina Infinium XT genotyping bead chip (Illumina, San Diego, CA, USA) commercially available as the MyDogDNA/Optimal Selection canine DNA test (Wisdom Health, Vancouver, WA, USA) (Donner et al. 2016, 2018).

### SD-OCT imaging

To examine the retinal phenotype associated with the *IFT122* variant in greater resolution, SD-OCT imaging was performed using the Heidelberg Spectralis HRA + OCT instrument (Heidelberg Engineering, Heidelberg, Germany) in the Department of Ophthalmology, Helsinki University Hospital, Helsinki, Finland. Imaging was performed to a PRA affected dog aged 13 years with the homozygous c.3176G > A variant and a clinically healthy wild-type LH aged 11 years. Thicknesses of the whole retina (including the RPE) and the presumed photoreceptor cell layer (including the RPE, the outer nuclear layer, and the inner and outer segments of the photoreceptor cells) were compared between the case and the control dog.

### Additional clinical measurements

To examine any extra-ocular manifestations that the *IFT122*-variant might have, three PRA affected LHs (one male, two females, aged 12–13 years), homozygous for the *IFT122* variant, were recruited for further clinical examinations, including general examination, complete blood count, serum biochemistry, abdominal ultrasound and thoracic radiographs. In addition, blood gas analysis, serum symmetric dimethylarginine (SDMA) and urinalysis including urine protein/creatinine ratio were studied in two of these dogs.

### IFT122 expression in human tissues and cells

Promoter-level expression data measured by cap analysis of gene expression (CAGE) (Forrest et al. 2014) was downloaded from FANTOM5 SSTAR (Abugessaisa et al. 2016) (https://fantom.gsc.riken.jp/5/sstar/Main_Page). The normalized expression values of genes with multiple CAGE peaks (promoters; *IFT122* and *IFT140*) were summed across all the peaks. The RNA-sequencing data of LUHMES differentiation was obtained from and processed as described in (Lauter et al. 2020). Briefly, read counts were normalized by the sum of spike-in reads for each sample (Katayama et al. 2013) and divided by the minimum non-zero value (5.78 × 10^–6^).

## Results

### Slowly progressing late-onset PRA in LHs

Over the years, several LH owners and breeders have contacted us as their dogs have been diagnosed with PRA that is not explained by the previously reported autosomal recessive *PRCD* and cmr3 variants (Zangerl et al. 2006, 2010). We analyzed LH samples donated to our canine biobank at the University of Helsinki to find the genetic cause of this new type of PRA. An initial study cohort of 44 LHs was included in the study, with readily available phenotype information as comprehensive eye examinations had been carried out on each individual dog as a part of breeding programs and health check-ups. These examinations had revealed clinical findings compatible with generalized PRA in 10 LHs (five males, five females), as these dogs presented with bilateral, diffuse tapetal hyperreflectivity and vessel attenuation. Two of the 10 cases were only examined as young or middle-aged adults (at 1.9 and 5.1 years of age) when they exhibited only mild fundus changes and were therefore diagnosed to have “PRA suspected”. The remaining eight dogs were also examined later in life and had been diagnosed as “PRA affected” at an average age of 9.0 years (± SD 2.9). Typical early findings included night blindness and diffuse tapetal hyperreflectivity. Disease progression was slow as some of the affected dogs still had some visual capacity left at 13 years. All the cases were genotyped for the *PRCD* p.C2Y and the cmr3 (p.P463fs, p.G489V) variants (Zangerl et al. 2006, 2010). Of the 10 cases, all were wild-type for the *PRCD* variant, while four were wild-type and six heterozygous for the two cmr3 variants, excluding these recessive variants as the genetic cause of their phenotype. The rest of the initial cohort, 34 LHs (16 males, 18 females), were regarded as controls, as they were examined healthy at the age of 10 years or older. The two cmr3 variants were also screened in these control dogs, indicating 25 wild-type and nine heterozygous dogs.

### GWAS indicates a PRA locus on chromosome 20

**Fig. 1:**
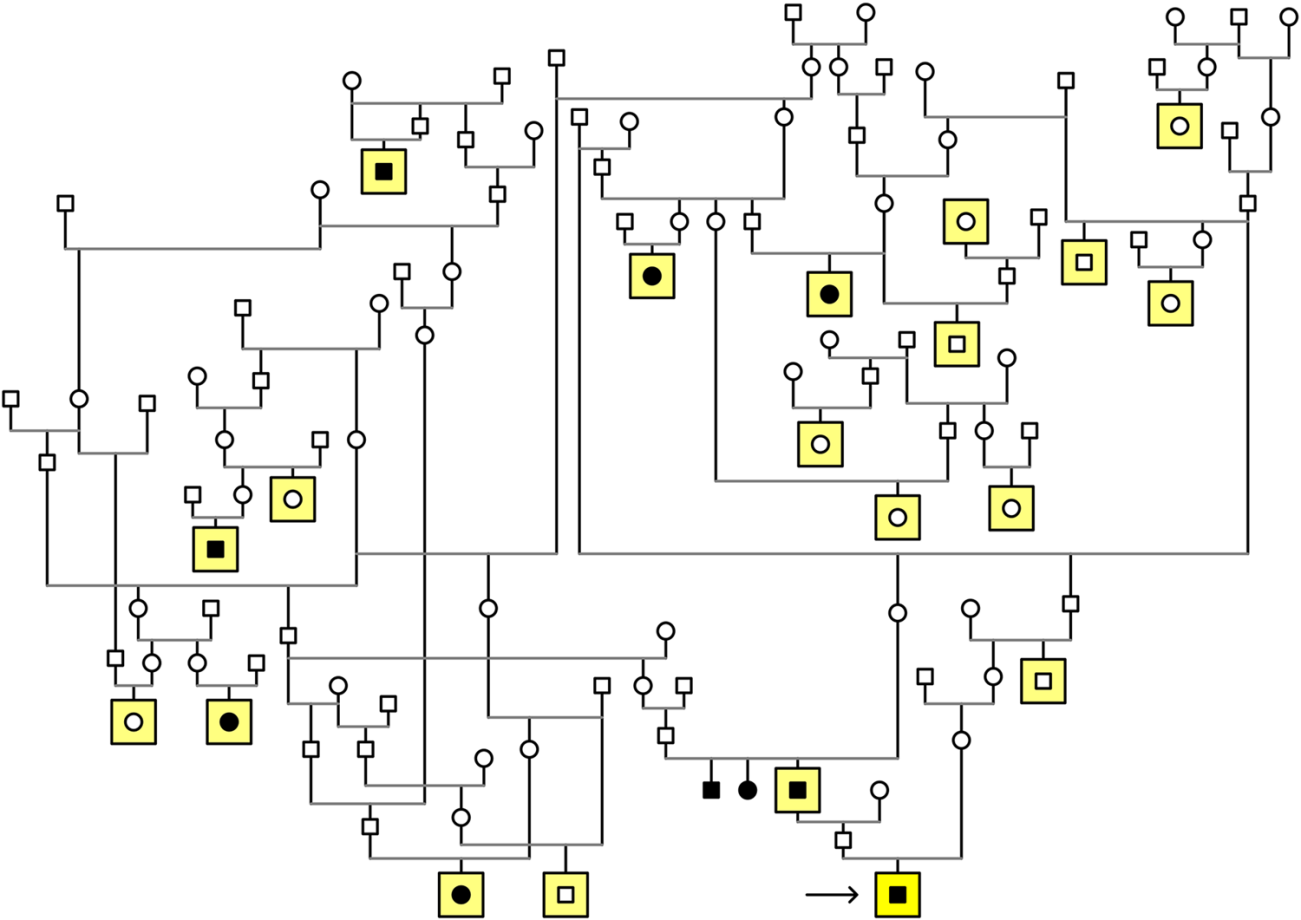
Pedigree analysis suggested autosomal recessive PRA in LH, as affected dogs were born to unaffected individuals, there were multiple cases in one of the affected litters, and both sexes were equally affected. The black symbol denotes affected dogs; squares indicate males and circles females, respectively. Dogs included in the GWAS are marked with a yellow background and the whole-genome sequenced case with a black arrow

We constructed a pedigree around the affected individuals to assess the mode of inheritance. This analysis suggested that the new type of PRA in LHs is likely to be an autosomal recessive condition, as the affected dogs were born to unaffected parents, there were multiple cases in one of the affected litters, and males and females were equally affected (Fig. 1).

**Fig. 2:**
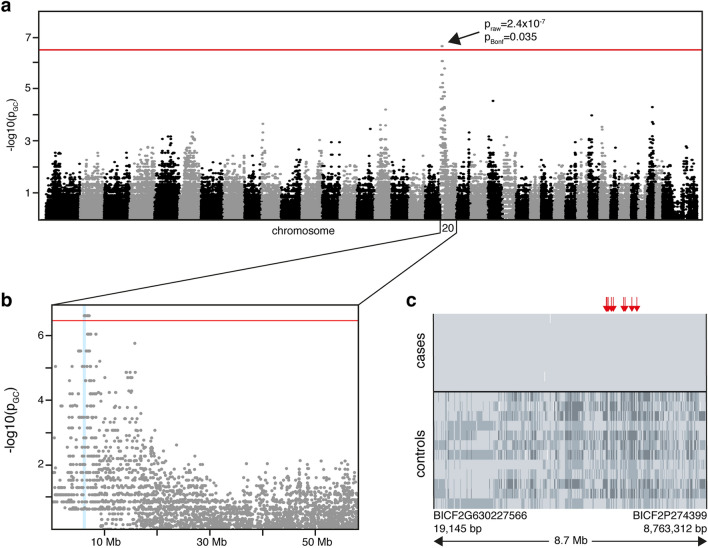
GWAS reveals a PRA locus on chromosome 20. **a**, **b** Results from the GWAS with 8 cases and 12 controls indicate a PRA locus on the canine chromosome 20 (*p*_raw_ = 2.4 × 10^–7^, *p*_Bonf_ = 0.035, *λ* = 1), where 9 markers meet genome-wide significance (threshold *p* = 3.4 × 10^–7^ marked with red line). **c** In-depth analysis of the genotypes in the associated locus reveals a shared homozygous haplotype block of 8.7 Mb in the cases absent in control dogs. Rows represent individual dogs and columns each SNP with light gray denoting affected genotypes, intermediate heterozygotes, and dark gray opposite homozygotes. The locations of the nine markers with genome-wide significance are marked with red arrows

We then performed a genome-wide association study (GWAS) with eight cases and 12 controls and with 145,387 single nucleotide polymorphism (SNP) markers to map the disease locus. Comparison of allele frequencies between cases and controls indicated a PRA associated locus on canine chromosome 20 with nine genome-wide significant markers (*p*_raw_ = 2.4 × 10^–7^, *p*_Bonf_ = 0.035, *λ* = 1, Fig. 2a, b). An in-depth analysis of the genotypes in the locus revealed an 8.7 Mb homozygous region at 19,145–8,763,312 bp in the affected LHs, not observed in the controls (Fig. 2c). As there were no informative markers upstream of the first homozygous marker at 19,145 bp, the subsequent variant search was performed from the beginning of CFA20 until the first heterozygous marker in a case at 8,894,743 bp.

### WGS reveals a recessive missense variant in IFT122

**Fig. 3:**
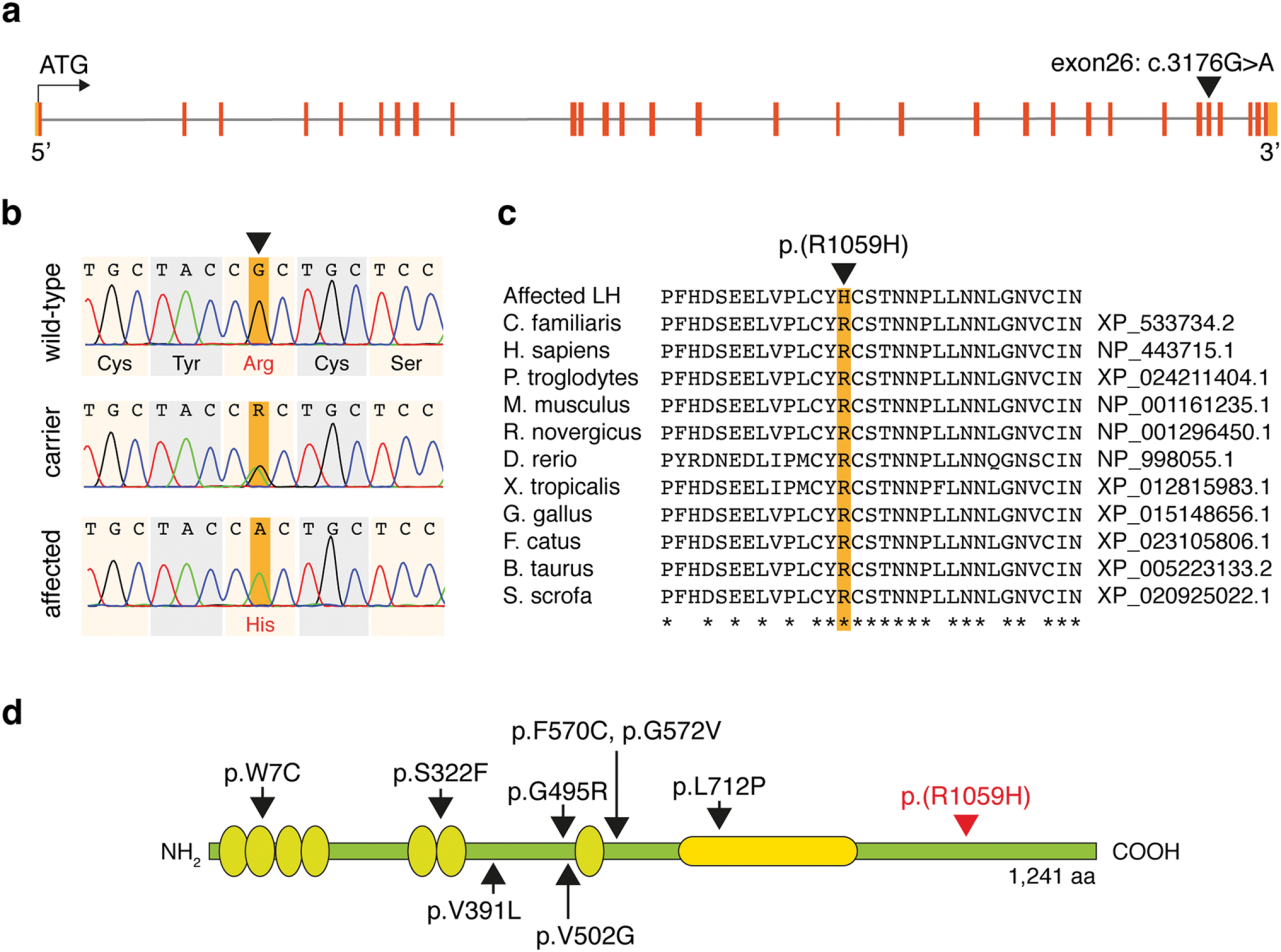
The *IFT122* sequence and protein structure. **a** Schematic presentation of the *IFT122* gene with exons marked in red, UTR sequences in orange and introns in gray. A missense variant c.3176G>A in exon 26 was identified in whole-genome sequencing data. **b** Chromatograms of the variant site (highlighted with orange background) and its surrounding sequence in wild-type, carrier and affected LH. **c** The variant site p.(R1059H) and its surrounding amino acids are highly conserved among different species. **d** Schematic presentation of the IFT122 protein with the canine PRA associated missense variant p.(R1059H) highlighted in red. The previously reported variants in human CED patients, all in relation to NP_443715.1, are marked with black arrows (Walczak-Sztulpa et al. 2010; Alazami et al. 2014; Tsurusaki et al. 2014; Moosa et al. 2016). The human IFT122 contains seven WD repeat domains, marked with light green circles, and a C-terminal TPR domain, marked with yellow

To find candidate variants in the associated chromosomal region, we next performed whole-genome sequencing on a PRA affected LH (Fig. 1, black arrow). In total, 498,114,393 reads were collected of which 99.37% were mapped to the reference sequence. The mean coverage was 30.3X. Comparing to the reference sequence, altogether 11,332 single nucleotide variants (SNV) and small deletions and insertions were homozygous in the associated chromosomal region. In addition, 49 mobile element insertions (MEIs) and 356 structural variants (SVs) were detected in the locus. After filtering against control dogs (Online Resource 1), 79 SNVs, one MEI, and one SV were case-specific (Online Resource 3). Only one non-synonymous variant was predicted to alter protein structure and was therefore chosen for additional screening: c.3176G>A in the intraflagellar transport 122-gene (*IFT122*, XM_533734.6, Fig. 3a, b) was predicted to lead to an amino acid substitution from arginine to histidine (p.R1059H, XP_533734.2). In silico analyses with PROVEAN and PolyPhen-2 predicted the variant to be deleterious (score = − 4.054, cut-off = − 2.5) and probably damaging (score = 0.961), respectively, which was in line with the Clustal Omega analysis showing conservation in the changed amino acid position (Fig. 3c). Both the dog and human IFT122 consist of 1,241 amino acids with 93% similarity at the amino acid level.

Validation of the IFT122 variant in the initial cohort of 10 PRA cases and 34 controls revealed complete segregation and full penetrance as all controls were either wild-type (*n* = 21) or heterozygous (*n* = 13), while all cases were homozygous (*n* = 10), supporting a recessive mode of inheritance. Subsequently, the variant was screened in all available LH samples in our biobank (*n* = 519), revealing 362 wild-type, 143 heterozygous and 14 homozygous LHs and a carrier frequency of 28%. Of the 14 new homozygotes, 6 had been diagnosed with PRA by veterinary ophthalmologists board-certified by the European College of Veterinary Ophthalmologists. Two dogs that were examined at the age of two and four years presented with tapetal hyperreflectivity. One dog had been eye examined at the age of eight years and diagnosed with mild cortical cataract. The owner reported a reduced vision in this dog but had thought it resulted from the cataract and did not take it to eye examination again before its death at 12 years of age. The remaining five dogs were not available for eye examinations, but a general veterinarian had suspected PRA in one dog. In addition, owners reported complete blindness in one dog while night blindness was reported in another dog. Of the 14 new *IFT122* variant homozygotes, 11 were wild-type and three heterozygous for the *PRCD* p.C2Y variant and eight wild-type and five heterozygous for the two cmr3 variants (p.P463fs, p.G489V), while DNA sample of one dog had run out and was not available for cmr3 testing.

### Breed-specificity of the IFT122 c.3176G>A variant

To study the breed-specificity of the IFT122 variant, we analyzed the genomic data available from additional 55 dogs from 16 breeds and 8 wolves available through the Dog Biomedical Variant Database Consortium (Online Resource 2). This analysis revealed one heterozygous Finnish Lapphund (FL), while other screened samples were wild-type.

To further study the segregation and prevalence of the variant in FLs, we genotyped samples from 577 FLs available in our biobank, including seven PRA affected dogs with the *PRCD* variant excluded. Of the tested dogs, 508 were wild-type, 68 heterozygous and 1 homozygous for the *IFT122* variant, indicating a carrier frequency of 12%. The only homozygote FL had been eye examined healthy at the age of five years, but no clinical information after that was available. Importantly, all the PRA affected FLs were wild-type for the *IFT122* variant, indicating that another genetic cause of PRA is evident in the breed.

To further investigate the breed-specificity of the variant, we gathered results from 6023 dogs from 183 breeds and 130 mixed breed dogs submitted for routine commercial gene panel screening at Genoscoper Laboratories Ltd, Helsinki during 2019–2020. These dogs were all wild-type for the variant.

### OCT imaging reveals retinal and photoreceptor cell layer thinning

**Fig. 4:**
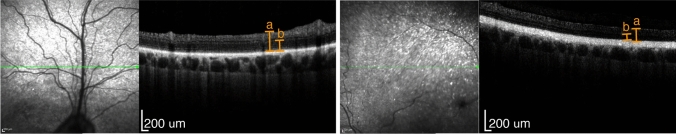
SD-OCT images of a healthy control (left-hand side) and a PRA affected *IFT122* variant homozygote (right-hand side) indicate reduced thickness of the whole retina and RPE (**a**) and the outer retinal layers, including the outer nuclear layer, inner and outer segments of photoreceptors, and RPE (**b**) in the affected dog (113 μm and 53 μm, respectively) compared to the control dog (185 μm and 92 μm, respectively)

To examine the retinal phenotype associated with the homozygous *IFT122* c.3176G > A variant in a greater detail, spectral-domain optical coherence tomography (SD-OCT) was performed for two dogs: a PRA affected, *IFT122* variant homozygote LH aged 13 years, and a clinically healthy wild-type LH aged 11 years. The image was acquired using the optic disc as a landmark. Due to limited visual perception, the affected dog could not fixate properly, and the image was obtained more peripherally. The thicknesses of the whole retina with the retinal pigment epithelium (RPE) and outer retinal layers, including outer nuclear layer, inner segments and outer segments of photoreceptors, and RPE, were severely reduced in the affected dog (113 μm and 53 μm, respectively) when compared to the control dog (185 μm and 92 μm, respectively) (Fig. 4). Interestingly, the photoreceptor layer was not completely lost despite the dog’s old age, supporting the observed slow progression rate in the other affected dogs, and also the owner’s assessment that the dog had reduced but not entirely lost vision.

### Clinical examinations to detect extra-ocular manifestations

IFT122 forms a part of the protein complex that regulates ciliary function (Rosenbaum and Witman 2002; Hirano et al. 2017; Takahara et al. 2018). Therefore, to understand the possible role of the IFT122 variant in extra-ocular manifestations, we performed clinical examinations including thoracic radiographs, abdominal ultrasound, complete blood count and serum biochemistry for three homozygous LHs (aged 12–13 years). All the three LHs had been diagnosed with generalized PRA. One of the females was completely blind, while the other two had reduced, but not totally lost ability to see. Apart from the ocular signs, all three dogs were clinically healthy. In laboratory tests, one of the dogs had mildly elevated creatinine value (118 μmol/l, reference < 116 μmol/l) and there was mild hematuria in that dog, while other renal parameters were normal in all of the dogs. Abdominal ultrasonography findings previously associated with *IFT122* variants in humans (Walczak-Sztulpa et al. 2010), including renal and liver abnormalities, were not detected. Thoracic radiographs were unremarkable in all dogs. None of the dogs had situs inversus.

### IFT122 expression in human

To explore the *IFT122* expression pattern across human tissues and cells, we utilized the Functional Annotation of Mammalian Genome 5 (FANTOM5) expression atlas, which catalogs promoter activities in a wide variety of human tissues and cells (Forrest et al. 2014). *IFT122* is most highly expressed in testis (Online Resources 4 and 5a). High expression is also observed in brain tissues, such as the pituitary gland, as well as the fetal kidney. Notably, it is also highly expressed in retina. As IFT122 forms the intraflagellar transport A (IFT-A) complex together with IFT144, IFT140, IFT43, IFT121 and IFT139 (Takahara et al. 2018), their expression levels were also studied and found to be generally comparable in retina, chondrocyte, and osteoblast (Online Resource 5b). We further investigated the expression changes of *IFT122* in Lund human mesencephalic (LUHMES) cell line which can recapitulate the neuronal cell development process in vitro (Lauter et al. 2020). Here we found that *IFT122* is strongly upregulated from day 2 to day 5 (Online Resource 5c), along with many other genes related to axonogenesis and ciliogenesis, indicating that IFT122 plays an important role in neuronal systems including retina.

## Discussion

Nonsyndromic RP is a significant cause of blindness in man, and over 70 genes and loci have been implicated in the disease (Daiger et al. 1998). However, the genetic background, in many cases, remains still unknown. We report here a recessive missense variant in *IFT122* as a candidate causal variant for a novel canine RP model and provide a new spontaneous large animal model to study the pathogenesis of RP and retinal biology.

*IFT122* encodes a cytoplasmic member of the WD-repeat protein family (Gross et al. 2001). IFT122 has seven N-terminal WD repeat domains and a C-terminal tetratricopeptide repeat domain (TPR) (Fig. 3d) (Takahara et al. 2018; Gross et al. 2001). Interaction in the TPR domain and IFT144 and IFT140 form the core subcomplex of the IFT-A complex, whereas the interaction between the WD repeat domain and IFT43 and IFT121 form its peripheral subcomplex together with IFT139 (Takahara et al. 2018). The core and the peripheral subcomplexes are connected by the interaction of IFT122, IFT43, and IFT121 (Takahara et al. 2018). IFT-A is essential for the assembly and maintenance of cilia as it mediates retrograde trafficking of ciliary proteins (Rosenbaum and Witman 2002; Hirano et al. 2017). The photoreceptor cells are initially formed from primary cilia, and in the adult photoreceptor cells, the outer and inner segments are connected to each other only by a connecting cilium (De Robertis 1956). The connection between these two segments is crucial for proper photoreceptor function, as the photopigments and light-transducing machinery are located in the outer segments, whereas protein synthesis occurs in the inner segments of the photoreceptor cells (Young 1976).

It is estimated that around 2,000 opsin molecules are required every minute to maintain the rod outer segments in mammals and the IFT machinery presumably has an important role in its transport (Rosenbaum and Witman 2002). Concordantly, studies on zebrafish larvae carrying a null mutation in *ift122* show that ift122 is essential for efficient opsin transport and the distal elongation of the outer segments (Boubakri et al. 2016). The mutant larvae presented with progressive photoreceptor degeneration with slow onset—a phenotype that resembles the disease in the affected LHs. To our knowledge, variants in *IFT122* have not previously been associated with RP in humans. Still, variants in *IFT144* and *IFT140*, the other two components of the IFT-A core subcomplex, have been implicated in both syndromic and nonsyndromic RP (Coussa et al. 2013; Hull et al. 2016). On the other hand, several variants in *IFT122* have been reported to cause cranioectodermal dysplasia (CED) in humans (Walczak-Sztulpa et al. 2010; Alazami et al. 2014; Tsurusaki et al. 2014; Moosa et al. 2016). The disorder, also called the Sensenbrenner syndrome, is characterized by craniofacial, skeletal and ectodermal abnormalities, but defects in liver, kidneys, teeth and skin have also been reported in patients with different *IFT122* variants (Walczak-Sztulpa et al. 2010; Alazami et al. 2014; Tsurusaki et al. 2014; Moosa et al. 2016). A report describing two CED-affected siblings without a molecular diagnosis also found evidence of photoreceptor dystrophy; the patients, examined at the age of four years, exhibited reduced scotopic and photopic responses in electroretinography, while fundus examination detected no abnormalities (Eke et al. 1996). These findings mimic the early phase of IFT122-PRA in LHs, where night blindness precedes ophthalmoscopic fundus changes. The lack of ocular signs in the CED patients with confirmed *IFT122* variants may be due to the affected site of the IFT122 protein. All but one of the reported variants in human patients reside in the N-terminal WD repeat domain, whereas the canine missense variant is located in the C-terminal region (Walczak-Sztulpa et al. 2010; Alazami et al. 2014; Tsurusaki et al. 2014; Moosa et al. 2016). Additionally, most of the CED affected individuals were examined only in early infancy, and the retinal dystrophy might develop later in life. As the *IFT122* was highly expressed in the human retina samples, we propose late-onset RP as a potential finding in the CED patients too.

In contrast to the IFT122-affected CED patients with skeletal abnormalities and other defects as well as to the *ift122* null zebrafish larvae model where the development of cystic kidneys was detected, similar extra-ocular manifestations were not observed in the LHs homozygous for the p.(R1059H) variant. The limited retinal phenotype in dogs may result from the affected site of the IFT122 protein or reflect species-specific differences in the IFT122 biology. We propose the former to be more plausible, as the discovered canine variant resides near the C-terminus, whereas all but one of the reported variants in CED patients are close to the N-terminal WD repeat domain (Fig. 3d). Interestingly, different variants in *IFT144*, another IFT-A core subcomplex component, have been reported to result both in syndromic and nonsyndromic RP (Coussa et al. 2013) and the same might also apply to *IFT122* variants. Furthermore, the canine *IFT122* variant is located in the C-terminal region that is known to interact with IFT144 (Takahara et al. 2018), and the functional IFT122-IFT144 interaction might be of particular importance for normal photoreceptor function. Only one of the reported variants in CED patients, the p.L712P, is located in the TPR domain (Moosa et al. 2016). The variant was found in compound heterozygous state from an Argentinian girl together with a splice-site variant in *IFT122* (Moosa et al. 2016). Retinal abnormalities were not reported, but seemingly, clinical data were available only from early infancy.

Additionally, studies on hTERT-RPE1 cells indicate that total abolition of IFT122 in knockout cells results in cilia absence, whereas even exogenous expression of CED-associated missense variants, located in the WD repeat domain, restored ciliogenesis, but not ciliary protein trafficking (Takahara et al. 2018). Therefore, we hypothesize that ciliary protein trafficking might be more susceptible to disturbances than ciliogenesis, and the IFT122 C-terminal interaction with IFT144 may be essential to protein, including opsin, trafficking in the photoreceptor cells. While we cannot make final conclusions of the canine *IFT122* variant’s exact pathological mechanism due to lack of suitable tissue material, it seems likely that the missense variant in the C-terminal region might lead to insufficient opsin transport in the photoreceptor cells leading to their degeneration, while ciliogenesis would remain normal. Previously, variants in cilia-associated genes *TTC8, BBS4, FAM161A* and *CCDC66* have been implicated in both syndromic and nonsyndromic PRA in several dog breeds (Dekomien et al. 2010; Downs et al. 2014; Downs and Mellersh 2014; Chew et al. 2017; Makelainen et al. 2020).

The *IFT122* variant reported here was also found in Finnish Lapphunds (FLs), a related breed, with a carrier frequency of 12%. Unfortunately, the only identified homozygous FL had been eye examined only at the age of five years and was not available for re-examination. As the average age at diagnosis in LHs was nine years, variant causality in the FLs remains to be determined. Amongst the screened 577 FLs there were seven PRA affected dogs with the known *PRCD* variant excluded, but all of them were wild-type for the *IFT122* variant. These results indicate that there is yet another genetic cause of PRA in FLs to be found.

In conclusion, we report here a missense variant in IFT122 as a likely cause of autosomal recessive PRA in LHs. Our study establishes a new spontaneous large animal model to study retinal biology and disease and implicates *IFT122* as a potential candidate gene for human RP. Further studies are needed to establish the exact pathological mechanism. Meanwhile, a gene test can be developed to aid veterinary diagnostics and breeding programs, which is of particular importance as the affected breed also suffers from other genetic forms of inherited retinal dystrophies (Zangerl et al. 2006, 2010).

## Supplementary Information

Below is the link to the electronic supplementary material.

Supplementary file1 Online Resource 1 List of the control dogs used for filtering the whole-genome sequencing data (numbers per breed) (XLSX 10 KB)

Supplementary file2 Online Resource 2 Non-LH samples tested for the IFT122 variant (XLSX 17 KB)

Supplementary file3 Online Resource 3 List of case-specific variants from the whole-genome sequenced affected LH (XLSX 16 KB)

Supplementary file4 Online Resource 4 Promoter-level expression of IFT122 in various human tissues and cells in FANTOM5 (XLSX 59 KB)

Supplementary file5 Online Resource 5 IFT122 expression pattern in human tissues and cells as well as neuronal differentiation process. a IFT122 expression in various human tissues and cell samples in FANTOM5. Samples are sorted from left to right in the order of the expression level from highest to lowest. b Expression of the six subunit genes of the IFT-A complex in retina, chondrocyte, and osteoblast. c IFT122 expression changes during the LUHMES cell differentiation. The error bars represent the standard error of the mean of replicates. tpm: tags per million (TIF 756 KB)

## Data Availability

WGS data from an affected LH have been submitted to the NCBI SRA under the accession number PRJNA682002 (BioProject), SAMN16976490 (BioSample).
