# Supplementary figures and images for: A missense variant in IFT122 associated with a canine model of retinitis pigmentosa
Source: Hum Genet. 2021 Feb 19;140(11):1569–79. doi: 10.1007/s00439-021-02266-3 (PMC8519925; doi:10.1007/s00439-021-02266-3)

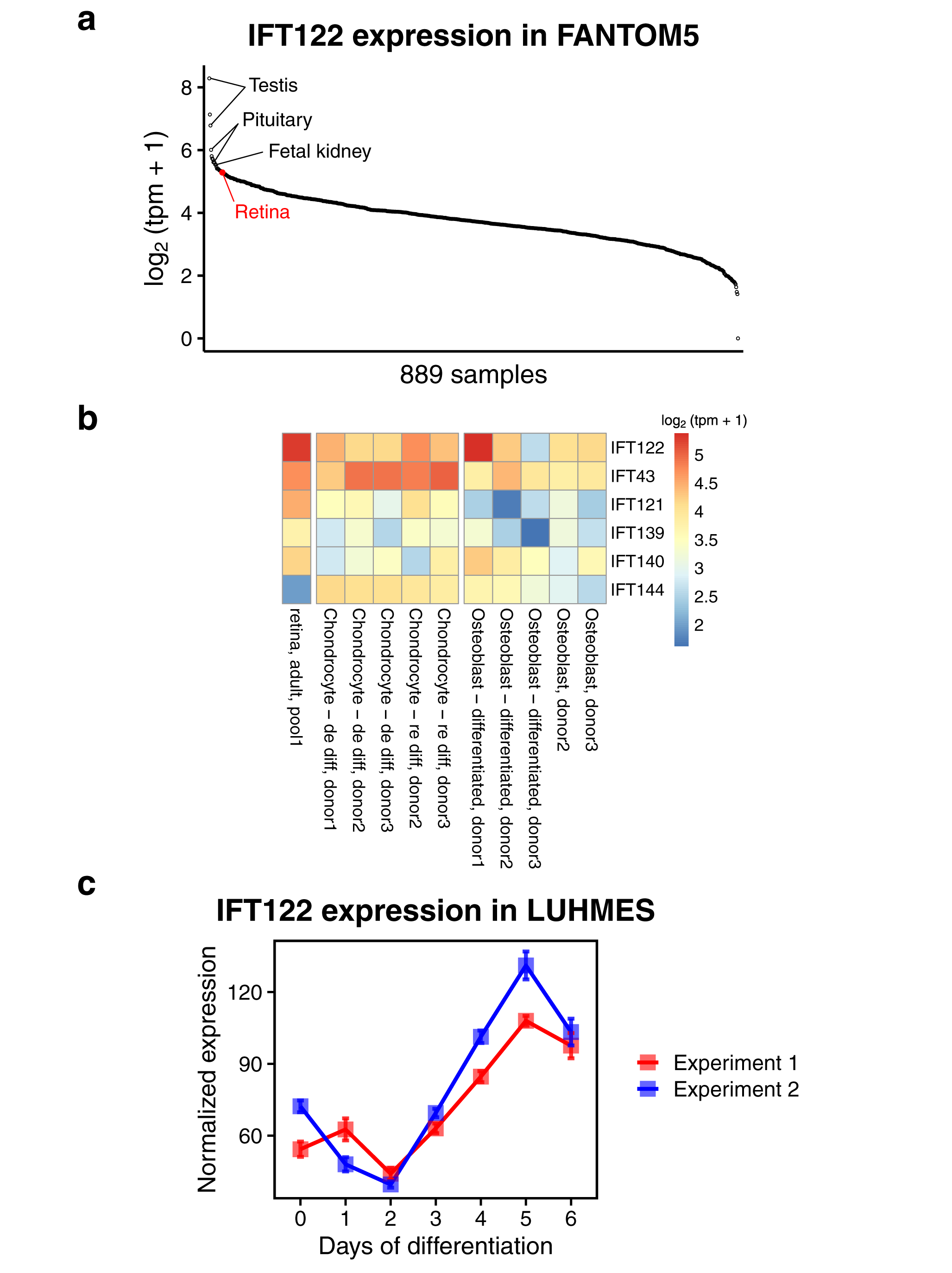

Supplement: Supplementary file 5 — Supplementary file5 Online Resource 5 IFT122 expression pattern in human tissues and cells as well as neuronal differentiation process. a IFT122 expression in various human tissues and cell samples in FANTOM5. Samples are sorted from left to right in the order of the expression level from highest to lowest. b Expression of the six subunit genes of the IFT-A complex in retina, chondrocyte, and osteoblast. c IFT122 expression changes during the LUHMES cell differentiation. The error bars represent the standard error of the mean of replicates. tpm: tags per million (TIF 756 KB) [file 439_2021_2266_MOESM5_ESM.tif]
